# Role of Calcium in Phosphatidylserine Externalisation in Red Blood Cells from Sickle Cell Patients

**DOI:** 10.1155/2011/379894

**Published:** 2010-09-26

**Authors:** Erwin Weiss, David Charles Rees, John Stanley Gibson

**Affiliations:** ^1^Department of Veterinary Medicine, University of Cambridge, Madingley Road, Cambridge CB3 0ES, UK; ^2^Department of Molecular Haematology, King's College School of Medicine, London SE5 9RS, UK

## Abstract

Phosphatidylserine exposure occurs in red blood cells (RBCs) from sickle cell disease (SCD) patients and is increased by deoxygenation. The mechanisms responsible remain unclear. RBCs from SCD patients also have elevated cation permeability, and, in particular, a deoxygenation-induced cation conductance which mediates Ca^2+^ entry, providing an obvious link with phosphatidylserine exposure. The role of Ca^2+^ was investigated using FITC-labelled annexin. Results confirmed high phosphatidylserine exposure in RBCs from SCD patients increasing upon deoxygenation. When deoxygenated, phosphatidylserine exposure was further elevated as extracellular [Ca^2+^] was increased. This effect was inhibited by dipyridamole, intracellular Ca^2+^ chelation, and Gardos channel inhibition. Phosphatidylserine exposure was reduced in high K^+^ saline. Ca^2+^ levels required to elicit phosphatidylserine exposure were in the low micromolar range. Findings are consistent with Ca^2+^ entry through the deoxygenation-induced pathway (P_sickle_), activating the Gardos channel. [Ca^2+^] required for phosphatidylserine scrambling are in the range achievable *in vivo*.

## 1. Introduction

Patients with sickle cell disease (SCD) display a range of symptoms which include chronic anemia together with ischemic pain and organ damage [1]. The underlying cause is the presence in patients' red blood cells (RBCs) of the abnormal hemoglobin, HbS [2]. HbS polymerises into rigid rods on deoxygenation, changing RBC shape from biconcave disc into the characteristic sickle appearance [3]. RBC membrane permeability is markedly abnormal [4] whilst HbS is also unstable, representing an oxidative threat [5]. Altered behaviour of these HbS-containing RBCs (here termed HbS cells), other circulating cells, and the endothelium combine to reduce RBC lifespan (hence the anemia) and also result in microvascular occlusion (hence the ischemia) [6]. Although the exact pathogenesis remains unclear, an important feature is considered to be increased exposure of phosphatidylserine (PS) on the outer bilayer of the RBC membrane [7–10]. Externalised PS is prothrombotic, and also provides a potential adhesion site for both macrophages and activated endothelial cells, contributing to both reduced HbS cell lifespan and vascular occlusion [11–13].

Two membrane phospholipid transporters represent the major determinants of PS exposure in RBCs: the ATP-dependent aminophospholipid translocase (APLT or flippase) transports aminophospholipids (APs), including PS, from outer to inner leaflet, whilst the Ca^2+^-dependent scramblase moves APs rapidly in both directions thus disrupting phospholipid asymmetry [14]. In normal RBCs, PS is largely confined to inner leaflet, through the dominant action of the flippase whilst the scramblase remains quiescent. A small, but variable, proportion of HbS cells from sickle cell patients, however, show exposure of PS ranging from about 2–10% [7, 9, 15, 16]. Both flippase inhibition and activation of the scramblase are probably involved [17]. Flippase inhibition could follow oxidative stress [18, 19], whilst scramblase activation could be caused by raised intracellular Ca^2+^ (e.g., [19, 20]) or other stimuli (e.g., [21]). The exact mechanisms, however, remain uncertain.

It is also well established that deoxygenation of HbS *in vitro* results in increased PS exposure [22, 23] but, again, the mechanism is not clear. Possibilities include disruption of the spectrin cytoskeleton [24], ATP depletion [25], decrease in intracellular Mg^2+^ [26], and also a rise in intracellular Ca^2+^ [20, 26]. In many reports concerning PS exposure, however, Ca^2+^ is not controlled or is present at unphysiological levels, making it difficult to assess its role definitively. In addition, whilst a more recent study correlated PS exposure in HbS cells with flippase inhibition, rather than elevation of intracellular Ca^2+^, the effects of deoxygenation were not determined [9]. 

Deoxygenation of HbS cells as well as causing HbS polymerisation and shape change, also activates a permeability pathway termed P_sickle_ [4, 27]. P_sickle_ is often described as a deoxygenation-induced cation conductance, apparently unique to HbS-containing red cells. A major importance of P_sickle_ is its permeability to Ca^2+^ [28, 29]. Although Ca^2+^ entry via this pathway represents an obvious link between HbS polymerisation and the deoxygenation-induced PS exposure, estimates suggest that the magnitude to which Ca^2+^ may be elevated is still relatively modest (around 100 nM) [29], and several orders of magnitude below that required for scramblase activation (around 100 *μ*M is usually cited [20, 30–32]). The present work is aimed at assessing the role of Ca^2+^ in PS exposure in RBCs from sickle cell patients.

## 2. Materials and Methods

### 2.1. Blood

Anonymised, discarded, routine blood samples (taken into the anticoagulant EDTA) were collected from individuals homozygous for HbS (HbSS genotype, *n* = 62) with approval from the local Ethics committee. After withdrawal, blood samples were kept refrigerated until used. (RBCs from HbSS individuals are here termed HbS cells).

### 2.2. Salines and Chemicals

HbS cells were washed into low (LK) or high potassium- (HK-) containing saline, comprising (in mM) NaCl 140, KCl 4, glucose 5, HEPES 10 for LK saline, and NaCl 55, KCl 90, glucose 5 and HEPES 10 for HK saline, all pH 7.4 at 37°C, with different extracellular [Ca^2+^]s ([Ca^2+^]_*o*_s) as indicated. When required, inhibitors (clotrimazole, DIDS, and dipyridamole) were added from stock solutions in DMSO. In these experiments, DMSO (final concentration 0.5%) was also added to controls. To investigate the effect of Ca^2+^ chelation, MAPTAM (5 *μ*M; Calbiochem, UK) was loaded into RBCs (5% haematocrit) for 60 min at 37°C with added pyruvate (5 mM) to prevent inhibition of glycolysis [33]. Extracellular chelator was removed by washing once with saline. Control RBCs without chelator were handled in the same way. FITC-labelled annexin V was obtained from Becton-Dickinson (Oxford, UK) in aqueous stock solutions (final concentration 0.3 *μ*g·mL^−1^). The calmodulin inhibitor *N*-(6-aminohexyl)-5-chloro-1-naphthalene sulphonamide (W-7) and the calcium fluorophore fluo-4-AM came from Invitrogen; all other reagents were obtained from Sigma (Poole, UK). 

### 2.3. Control of O_2_ Tension, Measurement of PS Exposure and Intracellular Ca^2+^

Salines and HbS cell suspensions were first equilibrated with humidified air (oxygenated) or N_2_ (deoxygenated) in Eschweiler tonometers (Eschweiler, Kiel, Germany). They were then placed in 24-well plates (10^8^ cells·mL^−1^, depth 3 mm) at 37°C in humidified incubators flushed with room air or 1% O_2_ (using a Galaxy-R oxygen incubator, RS Biotech, Irvine, UK) for 3–18 hours. After incubation, RBCs were treated with vanadate (1 mM) to inhibit flippase activity. They were then immediately harvested, washed once, and resuspended at a concentration of 5 × 10^6^ cells·mL^−1^ in binding annexin buffer (composition in mM: 145 NaCl, 2.5 CaCl_2_, 10 HEPES, pH 7.4) and incubated for 15 min at room temperature with FITC-labelled annexin (0.3 *μ*g·mL^−1^). Unattached annexin was then removed by washing once followed by resuspension in 5-times the initial volume of ice-cold binding buffer, after which samples were placed on ice. Percentage of RBCs with PS exposed on their external membrane was then measured in the FL-1 channel of a fluorescence-activated flow cytometer (FACSCalibur, BD), in which negative fluorescent gate was set using cells exposed to FITC-labelled annexin but in the absence of Ca^2+^ (which prevents annexin binding). PS exposure here refers to the percentage of RBCs which fluoresce more brightly than the negative gate. To alter intracellular [Ca^2+^], RBCs at 1% Hct were exposed to the calcium ionophore bromo-A23187 (1–6 *μ*M), vanadate (1 mM), EDTA (2 mM), and different [Ca^2+^]_*o*_s for 30 min to achieve the requisite final [Ca^2+^]_*o*_ [34]. This was multiplied by the square of Donnan ratio, *r* ^2^([H^+^]_*i*_/[H^+^]_*o*_)^2^ = 2.05 [35, 36], to calculate [Ca^2+^]_*i*_. After 30 min, RBCs were treated with Co^2+^ (0.4 mM, to block A23187) after which they were processed for annexin-labelling, as above. Annexin was used to label PS because it is important to compare findings with extensive reports in the literature using this PS marker (e.g., [8, 19, 37–39]). Bromo-A23187 (in preference to A23187 *per se*) was used because it does not fluoresce. These experiments were carried out in LK or HK saline, composition as above except for the addition of 0.15 mM MgCl_2_ to keep intracellular [Mg^2+^] at physiological levels. Finally, to show Ca^2+^-loading of RBCs, cells were loaded with fluo-4-AM (30 min at 37°C, 5 *μ*M; then washed once) with fluo-4 fluorescence also then measured in the FITC channel by FACS.

### 2.4. Statistics

Unless otherwise stated, data are presented as means ± S.E.M. for blood samples from *n* patients. Statistical significance of any differences was tested using paired Student's *t*-test (with *P* < .05 taken as significant).

## 3. Results

### 3.1. The Effect of Ca^2+^ on PS Exposure

**Figure 1:**
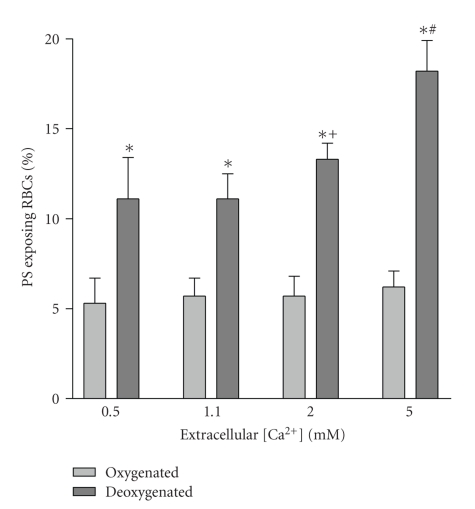
Effect of oxygen tension and extracellular Ca^2+^ on phosphatidylserine (PS) exposure in red blood cells (RBCs) from sickle cell patients. RBCs were incubated for 18 hours at four extracellular [Ca^2+^]'s (0.5, 1.1, 2.0 and 5.0 mM) after which they were labelled with FITC-annexin (as described in Section 2). Histograms representing mean percentage of positive RBCs ± S.E.M. for 5 different patients. *P < .01 deoxy compare to oxy; ^+^ *P* < .05 cf 0.5 mM Ca^2+^ deoxy; ^#^ *P* < .01 cf 0.5 mM Ca^2+^ deoxy.

**Figure 2:**
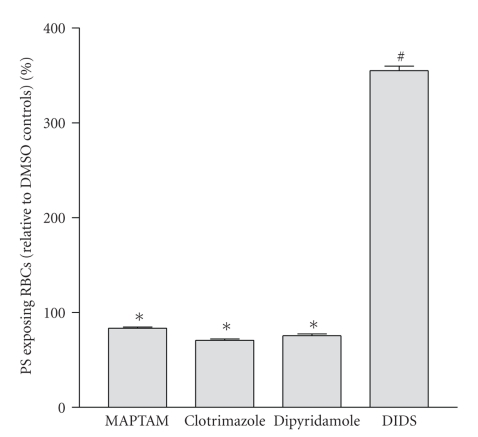
Effect of inhibitors on phosphatidylserine (PS) exposure in red blood cells (RBCs) from sickle cell patients. RBCs were incubated under deoxygenated conditions (1% O_2_) for 3 hours (5 mM extracellular [Ca^2+^]) after which they were labelled with FITC-annexin. Four conditions (all with 0.5% DMSO) are shown: MAPTAM-treated RBCs (loaded with 5 *μ*M MAPTAM prior to deoxygenation), clotrimazole (10 *μ*M), dipyridamole (50 *μ*M), and DIDS (50 *μ*M). Results are presented as percentage PS exposing RBCs relative to control RBCs exposed to 0.5% DMSO only. Histograms represent means ± S.E.M. (*n* = 3). *P < .01 and ^#^ *P* < .0001 cf DMSO controls.

PS exposure in HbS cell samples taken from SCD patients and immediately labelled with FITC-annexin ranged from 0.4 to 16.0% with a mean of 2.3 ± 0.5% (*n* = 36). The effect of different [Ca^2+^]_*o*_s (0.1, 0.5, 1.1, 2 and 5 mM) on the percentage of HbS cells showing PS exposure was then investigated. In oxygenated (20% O_2_) HbS cells, PS exposure was lower and although the extent of exposure was augmented when RBCs were incubated at higher [Ca^2+^]_*o*_s, the effect was small and not significant (Figure 1). When cells were deoxygenated (1% O_2_), PS exposure was always higher than that observed in oxygenated HbS cells. There was also a marked increase in PS at the higher [Ca^2+^]_*o*_s (Figure 1). This effect was present within 30 min, with longer incubation periods increasing the effect. To determine whether Ca^2+^ was acting extracellularly or intracellularly, HbS cells were loaded with the Ca^2+^ chelator MAPTAM prior to deoxygenation (Figure 2). Over a 3 hour period, MAPTAM decreased the percentage of positive HbS cells (*P* < .01). This inhibitory effect did not persist over an 18 hour incubation, probably because the available cytoplasmic MAPTA becomes saturated with Ca^2+^.

### 3.2. Effect of Partial P_sickle_ Inhibitors on PS Exposure

Although there are no specific inhibitors of P_sickle_, dipyridamole is partially effective [40]. When present during deoxygenation, dipyridamole (50 *μ*M) reduced PS exposure in deoxygenated HbS cells (Figure 2; *P* < .01), consistent with Ca^2+^ entry via P_sickle_ stimulating exposure. DIDS, although better known as a band 3 inhibitor, is also a partial P_sickle_ inhibitor [41]. Addition of DIDS (50 *μ*M), however, produced a marked increase in PS exposing RBCs with percentage of positive RBCs increasing several folds (Figure 2; *P* < .01). When DIDS was added to RBCs from normal HbAA individuals, PS exposure was also similarly increased: to 95.0 ± 0.3% in oxygenated conditions, and to 98.7 ± 0.1% in deoxygenated cells (both means ± S.E.M., *n* = 3). These findings suggest that annexin binding was caused by DIDS reacting with its target on the RBC membrane. HbS cells exposed to DIDS, but not subsequently treated with FITC-annexin, did not fluoresce (e.g., 0% DIDS-treated without FITC-annexin cf 50% DIDS-treated with annexin), indicating that the high values were not due to fluorescence from DIDS itself.

### 3.3. PS Exposure and Red Cell Shrinkage

**Figure 3:**
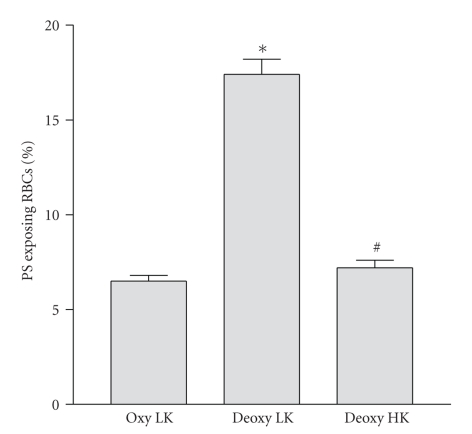
Effect of extracellular K^+^ on phosphatidylserine (PS) exposure in red blood cells (RBCs) from sickle cell patients. RBCs were incubated for 18 hours with extracellular [Ca^2+^] of 5 mM under oxygenated (20% O_2_) or deoxygenated (1% O_2_) conditions in either low K^+^-containing (extracellular [K^+^] of 5 mM) saline or high K^+^-containing (90 mM [K^+^]) saline. Histograms represent means ± S.E.M. (*n* = 3). *P < .001 compare to LK oxy; ^#^N.S. cf. LK oxy.

Elevated intracellular Ca^2+^ activates the Gardos channel and leads to K^+^ loss with Cl^−^ following through separate Cl^−^ channels [4]. PS exposure could therefore be secondary to the ensuing cell shrinkage [37]. To investigate this possibility, HbS cells were suspended in high K^+^-containing saline (90 mM) to remove any gradient for K^+^ efflux. The deoxygenation-induced increase in PS exposure was abolished (Figure 3), with values reduced to those observed in oxygenated samples (*P* < .001 deoxy LK cf oxy LK; N.S. deoxy HK cf oxy LK). An estimate of RBC size is provided by FACS forward scatter measurement. Forward scatter was 487 ± 8 (means ± S.E.M., *n* = 3) in oxygenated LK saline, falling to 439 ± 4 in deoxygenated LK saline (*P* < .005). In deoxygenated HK saline a value of 497 ± 3 was obtained (N.S. cf. oxygenated LK saline). PS exposure following deoxygenation in LK saline was therefore accompanied by cell shrinkage. This was not observed during deoxygenation in high K^+^ saline. A second method of inhibiting the Gardos channel, treatment with clotrimazole (10 *μ*M), was also tested. In this case, however, PS exposure was only partially prevented (Figure 2; *P* < .01). 

### 3.4. PS Exposure and Direct Manipulation of Intracellular [Ca^2+^]

**Figure 4:**
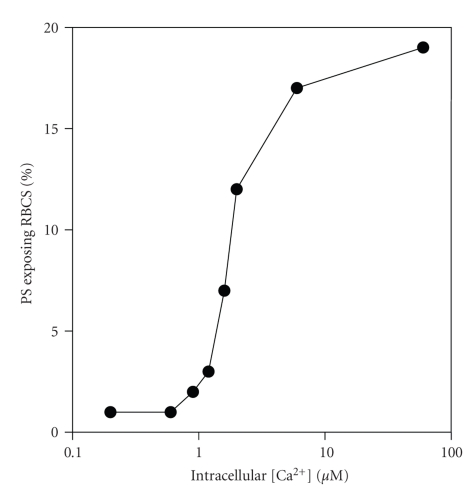
Effect of manipulation of intracellular Ca^2+^ on phosphatidylserine (PS) exposure in red blood cells (RBCs) from sickle cell patients. RBCs were first treated with vanadate (1 mM) to inhibit the plasma membrane Ca^2+^ pump and also the aminophospholipid translocase (flippase) before addition of bromo-A23187 (1.2 *μ*M, 1% haematocrit) and requisite extracellular [Ca^2+^]s for 30 min. They were then treated with Co^2+^ (0.4 mM) before labelling with FITC-annexin. Intracellular [Ca^2+^] is calculated from extracellular [Ca^2+^] × *r* ^2^, where *r* ^2^ was taken as 2.05 [36]. Results presented are from a single experiment representative of 5 others.

Treatment of RBCs with the divalent cation ionophore bromo-A23187 was used to alter intracellular [Ca^2+^] directly [34, 35]. RBCs were initially treated with vanadate (1 mM), to inhibit both the plasma membrane Ca^2+^ pump and also the flippase. Following 30 min incubation with bromo-A23187 to alter [Ca^2+^]_*i*_, Co^2+^ (0.4 mM) was then added to block Ca^2+^ permeability via A23187 thereby keeping intracellular [Ca^2+^] constant during annexin labelling (for which 2.5 mM extracellular [Ca^2+^] is required). Results are shown in Figure 4. PS exposure is elicited as [Ca^2+^]_*i*_ increased above about 600 nM. A sigmoidal dependence of PS exposure with [Ca^2+^] was then apparent with an EC_50_ of 1.31 ± 0.84 *μ*M (*n* = 6). Peak exposures varied from 16–46%, mean 28 ± 5 (*n* = 6) with a plateau reached at about 10 *μ*M and without further change at higher [Ca^2+^]_*i*_s ([Ca^2+^]s up to 600 *μ*M were tested).

### 3.5. Modulation of PS Exposure

**Figure 5:**
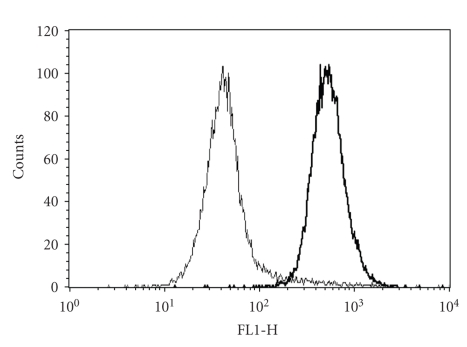
Ca^2+^ loading of red blood cells (RBCs) from sickle cell patients. RBCs were loaded with the Ca^2+^ fluorophore fluo-4 (see Methods). They were then incubated for 30 min in the absence (left—thin line) or presence (right—thick line) of bromo-A23187 at an extracellular [Ca^2+^] of 1 *μ*M. Results are presented as histogram of fluorescence of a single experiment representative of 3.

**Figure 6:**
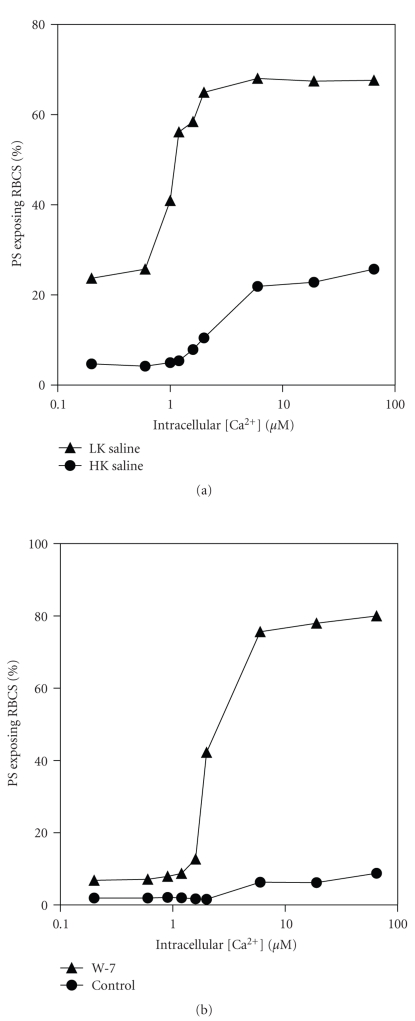
Effect of K^+^ and calmodulin inhibition on Ca^2+^-induced exposure of phosphatidylserine (PS) in red blood cells (RBCs) from sickle cell patients. Experimental details were as described in the legend to Figure 4, except that in (a) where incubation was carried out in either high K^+^-(HK, K^+^ = 90 mM) or low K^+^-containing saline (LK, 4 mM), and, in (b) where HK saline was used in the absence or presence of W-7 (100 *μ*M). Results are presented as single experiments representative of 3 others.

In the preceding section, although high affinity Ca^2+^-induced scrambling was present, it was noticeable that nevertheless only a minority of all RBCs stained positively for PS using FITC-annexin—as is also found in many literature reports, for example, [39]. That Ca^2+^ loading was complete and homogeneous was first ascertained using intracellular fluo-4 (Figure 5). It is apparent that the majority of RBCs (98 ± 1%, *n* = 3) were Ca^2+^-loaded. Uneven Ca^2+^ loading can therefore be discounted. As K^+^ has been reported to inhibit PS scrambling [42], the effect of 30 min incubation in LK saline compared to HK was determined in the presence of bromo-A23187 and different [Ca^2+^]. LK saline was found to increase the percentage of positive cells (Figure 6(a)), an effect again partially inhibited by clotrimazole (10 *μ*M) which, for example, reduced percentage of positive cells from 44% to 28% at 10 *μ*M Ca^2+^. Finally, the effect of the calmodulin inhibitor W-7 was tested (Figure 6(b)). In this case, the percentage of positive RBCs increased. It was noticeable, however, that in all these manoeuvres, Ca^2+^ affinity was unaffected (Figure 6).

## 4. Discussion

Whilst it is well known that RBCs from SCD patients show elevated levels of PS exposure and that these are increased upon deoxygenation, the mechanism is not clear. The present results explore more fully that the role of Ca^2+^. Ca^2+^ concentrations required for scrambling is considerably lower than previously appreciated. The Ca^2+^ affinity of the scrambling process is not dissimilar to that associated with inhibition of flippase activity or activation of the Ca^2+^-activated K^+^ channel (Gardos channel). This important finding suggests coordination of these eryptotic events. Results also implicate a role for RBC shrinkage and shape change.

### 4.1. Role of Ca^2+^ and P_sickle_ on PS Exposure

Altering extracellular Ca^2+^ levels had little effect on PS exposure in oxygenated HbS cells. Under deoxygenated conditions, however, PS exposure increased with [Ca^2+^]_*o*_. This effect was partially inhibited by dipyridamole [40] and by intracellular Ca^2+^ chelation with MAPTAM treatment [34]. These findings are consistent with Ca^2+^ entering via the deoxygenation-induced pathway P_sickle_ [4, 27] and acting intracellularly. Intracellular Ca^2+^ can have several actions. First, it will activate the Gardos channel leading to RBC shrinkage [43]. Second, it may stimulate the Ca^2+^-dependent scramblase whilst inhibiting the ATP-dependent flippase [14]. Third, it may stimulate cysteine proteases [44]. Any of these events may lead to PS exposure [21]. Several manoeuvres were tested to separate these possibilities. The most effective way of inhibiting PS exposure was incubation in high K^+^ saline. Removal of the electrochemical gradient for K^+^ efflux abolished the deoxygenation-induced increase in PS exposure. The Gardos channel inhibitor clotrimazole also partially inhibited PS exposure. Findings are consistent with the hypothesis that activation of P_sickle_, by deoxygenation mediates Ca^2+^ entry, elevating [Ca^2+^]_*i*_ which then promotes PS exposure by Gardos channel activation, loss of intracellular solutes, and red cell shrinkage. Importantly, high K^+^ salines were effective over all incubation times (up to 18 hours). Shrinkage has been shown previously to stimulate PS exposure in both normal RBCs and HbS cells [37, 45] and would appear to be involved in deoxygenation-induced PS exposure in sickle cells.

### 4.2. Ca^2+^ Dependence of PS Exposure

A major aim of this work was to determine unequivocally the intracellular Ca^2+^ required to elicit PS exposure in HbS cells. This was investigated using RBCs loaded with different [Ca^2+^]s using bromo-A23187. RBCs were first treated with vanadate (to inhibit both the plasma membrane Ca^2+^ pump and the flippase) and subsequently with Co^2+^ (which blocks A23187 so that the relatively high [Ca^2+^] required for annexin binding, 2.5 mM, could not gain access to the cytoplasm). Results showed that PS exposure was stimulated by micromolar Ca^2+^ concentrations with an EC_50_ of about 1.2 *μ*M. This concentration is similar, though slightly higher, compared with that required for half-maximal activation of the Gardos channel activation [46, 47] and for inhibition of the flippase [26]. A similar high affinity for Ca^2+^ was also observed in RBCs incubated in LK saline indicating that high K^+^ levels are not responsible for these observations. Calmodulin is known to interact with RBC cytoskeleton and influence PS exposure [48, 49]. Incubation with the calmodulin antagonist W-7 again showed a similar high Ca^2+^ affinity for PS exposure. In this case, the percentage of positive cells was also increased so that the majority of RBCs became positive, showing that most RBCs are capable of PS scrambling at these low Ca^2+^ levels. Previously reported values for activation of the scramblase are considerably higher than those given here, with values of 25–100 *μ*M quoted [14, 32]. Previous measurements, however, were made largely on resealed RBC ghosts, inside-out vesicles, or purified PLSCR1 [30, 31, 50, 51], which may not in fact represent the RBC scramblase [52]. These preparations will also necessarily lack much of the cytoplasmic contents which may result in reduction in Ca^2+^ affinity of the scrambling process. Furthermore, several previous reports were carried out in the presence of high concentrations of extracellular Mg^2+^ (1 mM) [20, 30, 50], which with the ionophore A23187 would set intracellular Mg^2+^ at over 2 mM, considerably in excess of the normal RBC [Mg^2+^] [53], and which might be expected to dampen any Ca^2+^ driven process. We speculate that having a similar Ca^2+^ level for Gardos channel activation, flippase inhibition and activation of scrambling would coordinate eryptotic events [21] and facilitate removal damaged RBCs in normal individuals, whilst in SCD patients, hyperactivity of these processes may contribute to disease pathogenesis.
